# Bilateral Skull Fractures in an Infant: Diagnostic Dilemma Between Accident and Abuse

**DOI:** 10.1155/crpe/4492559

**Published:** 2026-05-18

**Authors:** Tomohiro Hirade, Daisuke Koike

**Affiliations:** ^1^ Department of Pediatrics, Shimane Prefectural Central Hospital, Shimane, Japan, spch.izumo.shimane.jp

**Keywords:** abusive head trauma, accidental head trauma, bilateral skull fractures, Child Protective Services, scalp swelling

## Abstract

Accidental head injury in infants is relatively common in the emergency department. However, abusive head trauma (AHT) should be considered in the differential diagnosis because of its strong association with morbidity and mortality. A 4‐month‐old male infant was admitted to the emergency department by his parents because of bilateral scalp swelling. Skull radiography and head computed tomography with three‐dimensional reconstruction revealed bilateral linear parietal skull fractures. Although bilateral skull fractures are more commonly associated with AHT than single linear skull fractures, they may also result from a single accidental impact. Because skull fracture patterns alone cannot exclude AHT, a comprehensive medical evaluation is essential. If clinicians have even a slight suspicion of AHT, they should not hesitate to consult the hospital child protection team or Child Protective Services.

## 1. Introduction

Accidental head injury in infants is relatively common in the emergency department. However, abusive head trauma (AHT) should be considered in the differential diagnosis of apparent accidental head injury because of its strong association with morbidity and mortality [1]. AHT is defined as an injury to the skull or intracranial contents of an infant or young child due to inflicted blunt impact and/or violent shaking. The incidence of AHT is estimated at 25 to 35 per 100,000 children annually during the first year of life [2]. The early detection of AHT is vital to prevent escalating child abuse and save lives. To facilitate the early recognition and identification of AHT, specific features of patient’s history, physical examination, laboratory studies, and radiologic findings have been demonstrated. However, some cases with accidental head injury in infants are difficult to differentiate from AHT. Here, we report an infant case of bilateral skull fractures.

## 2. Case Report

A 4‐month‐old male infant was admitted to the emergency department by his parents because of bilateral scalp swelling. His parents stated that the father had accidentally dropped him onto a hard surface while holding him from a height of approximately 1.2 m, 1 week prior. The parents did not take their son to the hospital because he cried immediately after the fall and had no other symptoms. However, because of the gradual swelling of the bilateral scalp, he was admitted to the emergency department.

**FIGURE 1:**
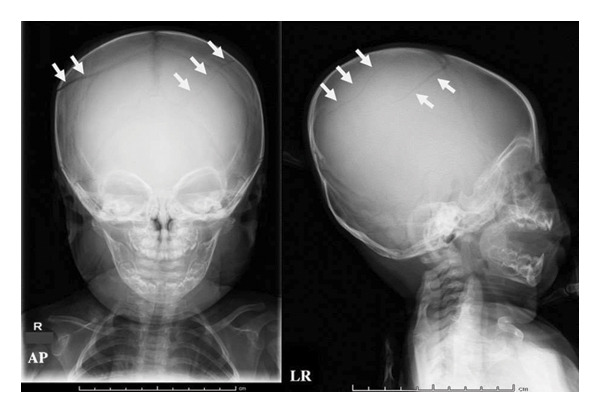
Skull radiography shows bilateral linear skull fractures (white arrows).

**FIGURE 2:**
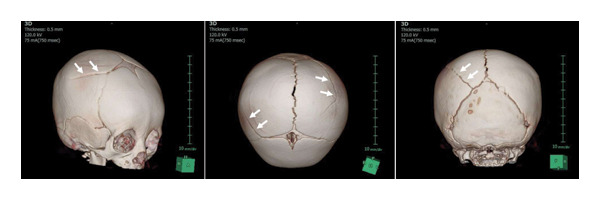
Head three‐dimensional computed tomography clearly shows linear biparietal skull fractures (white arrows).

On admission, the infant was in good general condition. Physical examination revealed bilateral scalp swelling over the parietal regions without additional findings such as bruising, burns, or vomiting. Skull radiography revealed bilateral linear skull fractures (Figure 1). Head computed tomography (CT) with three‐dimensional reconstruction clearly demonstrated bilateral linear parietal skull fractures (Figure 2). Magnetic resonance imaging identified a small epidural hemorrhage and confirmed the presence of a scalp hematoma. No additional injuries were detected on a whole‐body skeletal survey, ophthalmologic fundus examination, or laboratory screening for visceral injury. However, AHT could not be excluded because of the bilateral skull fractures and delayed presentation to medical services. We held a meeting with a child protection team in our hospital and reported the incident to Child Protective Services (CPS).

After CPS interviewed the parents, they concluded that the fractures were a result of an accidental fall because there were no inconsistencies in the parents’ description, no prior history of abuse, and no additional physical or radiological findings indicative of abuse. The infant was discharged after 2 days of hospitalization and continued follow‐up as an outpatient.

## 3. Discussion

Accidental head injury resulting from a fall is a leading cause of emergency department presentation in infants and young children. The most common fall height from which skull fractures occur accidentally is between 1 and 1.5 m [3]. Linear skull fractures, particularly linear parietal skull fractures, are common in both accidental head trauma and AHT. Isolated single linear skull fractures are less likely to be associated with AHT [4, 5]. In contrast, bilateral skull fractures have been reported to be more strongly associated with AHT than single linear skull fractures [5, 6]. However, recent studies suggest that bilateral simple linear skull fractures occur more frequently in accidental head trauma than previously appreciated [7, 8]. In addition to drop tests using infant cadavers and animal models, finite element analyses have demonstrated that bilateral skull fractures can result not only from double‐impact or compressive trauma (e.g., falls down stairs or television tip‐overs) but also from a single impact [9]. Because the infant skull is thin and pliable with low structural rigidity, relative movements of the skull plates are expected to cause fractures away from the impact site. In particular, occipital impacts have the potential to cause parietal skull fractures in falls from approximately 1 m onto hard surfaces [9]. In contrast, more complex skull fracture patterns, including branching, comminuted, depressed, stellate, ping‐pong, and skull fractures crossing cranial sutures or contacting two or more cranial sutures, are reported more frequently in AHT [7, 10]. Accordingly, fracture morphology should be carefully evaluated in conjunction with clinical findings.

In the present case, head CT with three‐dimensional reconstruction clearly delineated the bilateral linear parietal skull fractures. Compared with radiography and conventional two‐dimensional CT, three‐dimensional CT reconstruction enhances visualization of subtle fractures and improves differentiation from normal anatomical variants [11, 12].

Scalp swelling is frequently observed with isolated skull fractures and is a common reason for caretakers to bring their children to the hospital. However, as scalp swelling attributed to skull fractures often swells gradually within a week, minor accidental skull fractures result in delayed medical care seeking [2]. Consequently, delayed presentation is not uncommon and should not automatically be interpreted as evidence of neglect. Furthermore, scalp swelling overlying skull fractures can be mistaken for the impact site because scalp swelling is not always observed at the impact site. A recent study suggested that infants presenting with isolated scalp hematoma more than 24 h after head injury had good outcomes but a high prevalence of underlying injury on imaging [13]. Although subdural hemorrhages are high risk of AHT, epidural hemorrhages most often occur from low‐height/short‐distance falls and are associated with skull fractures [14].

Infants presenting with bilateral skull fractures, particularly following unwitnessed trauma, require careful and detailed evaluation for AHT. Because skull fracture patterns and scalp swelling alone cannot exclude AHT, a comprehensive evaluation should include detailed history taking, thorough physical examination, appropriate radiologic and laboratory studies, and ophthalmologic consultation. Even upon a slight suspicion of AHT, clinicians should not hesitate to consult the hospital child protection team or CPS. If clinicians misdiagnose patients or do not report to CPS, children with AHT will be at an increased risk of recurrent abuse, with possible traumatic neurological consequences [15, 16].

## 4. Conclusion

Although bilateral skull fractures are more commonly associated with AHT than isolated single linear skull fractures, they may also result from a single accidental impact. Scalp swelling attributed to skull fractures may develop gradually. Consequently, minor accidental skull fractures tend to delay medical care seeking. Because skull fracture patterns alone cannot exclude AHT, comprehensive clinical evaluation of AHT should be performed. If clinicians have even a slight suspicion of AHT, they should not hesitate to consult the hospital child protection team or CPS.

## Ethics Statement

In accordance with institutional policy, ethical approval is not required for anonymized case reports.

## Consent

Written informed consent was obtained from the parents for publication of the case report.

## Conflicts of Interest

The authors declare no conflicts of interest.

## Data Availability

The data that support the findings of this study are available from the corresponding author upon reasonable request.
